# Recent Applications of the (TMS)_3_SiH Radical-Based Reagent

**DOI:** 10.3390/molecules17010527

**Published:** 2012-01-06

**Authors:** Chryssostomos Chatgilialoglu, Jacques Lalevée

**Affiliations:** 1 ISOF, Consiglio Nazionale delle Ricerche, 40129 Bologna, Italy; 2 Institut de Science des Matériaux de Mulhouse IS2M, LRC CNRS 7228-ENSCMu-UHA, 15, rue Jean Starcky, 68057 Mulhouse Cedex, France; Email: Jacques.Lalevee@uha.fr

**Keywords:** tris(trimethylsilyl)silane, radical reactions, silyl radical, reduction, hydrosilylation, photopolymerization

## Abstract

This review article focuses on the recent applications of tris(trimethylsilyl)silane as a radical-based reagent in organic chemistry. Numerous examples of the successful use of (TMS)_3_SiH in radical reductions, hydrosilylation and consecutive radical reactions are given. The use of (TMS)_3_SiH allows reactions to be carried out under mild conditions with excellent yields of products and remarkable chemo-, regio-, and stereoselectivity. The strategic role of (TMS)_3_SiH in polymerization is underlined with emphasis on the photo-induced radical polymerization of olefins and photo-promoted cationic polymerization of epoxides.

## 1. Introduction

In the late eighties, Chatgilialoglu and coworkers introduced tris(trimethylsilyl)silane, (TMS)_3_SiH, as a radical-based reducing agent for functional group modifications and a mediator of sequential radical reactions [1]. (TMS)_3_SiH has found multiple applications in organic synthesis as well as in polymers and material science [2,3,4,5]. The purpose of this article is to give some recent examples of the use of (TMS)_3_SiH in radical chemistry.

## 2. (TMS)_3_SiH as Radical-Based Reducing Agent

**Scheme 1:**
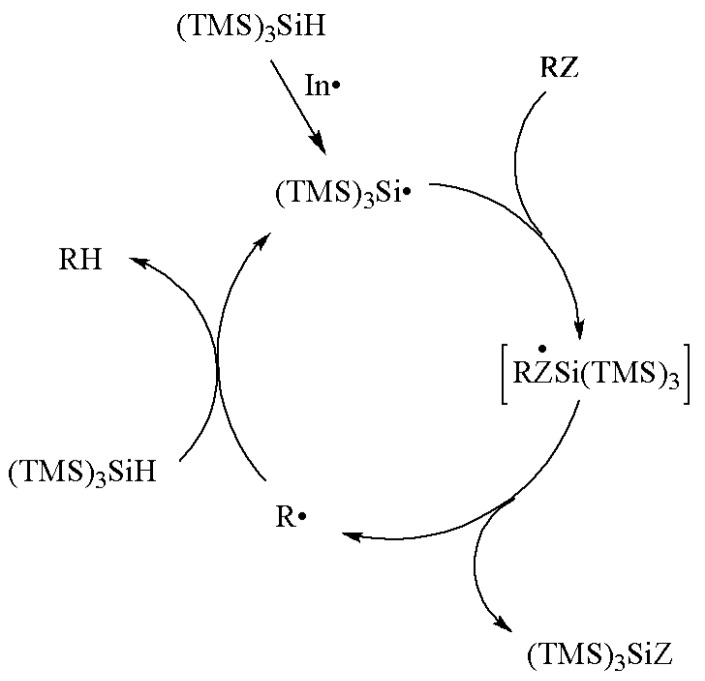
Mechanism of the reduction of a functional group by (TMS)_3_SiH.

**Scheme 2:**
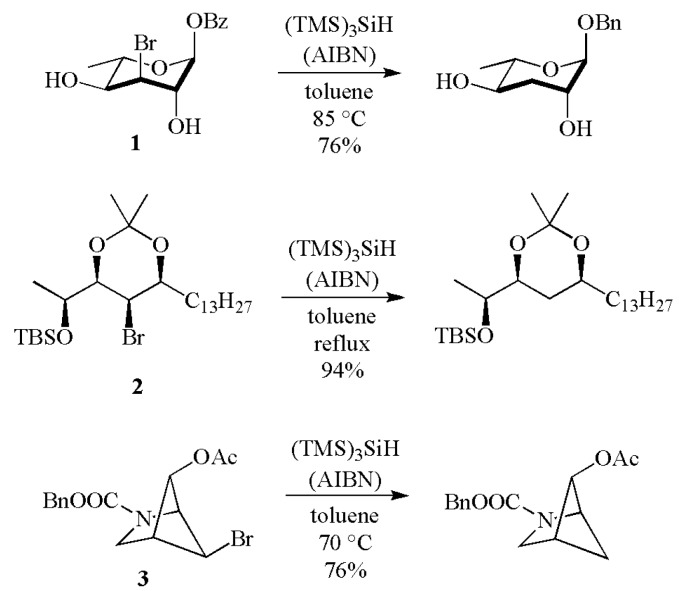
Debromination by reaction with (TMS)_3_SiH.

The majority of radical reactions of interest to synthetic chemists are chain processes under reductive conditions. The mechanism of the reduction of a functional group by (TMS)_3_SiH is shown in Scheme 1 [1,2,3,4,5]. Initially, (TMS)_3_Si^•^ radicals are generated by some initiation process. In the propagation steps, the removal of the functional group Z in the organic substrate (RZ) takes place by action of (TMS)_3_Si^•^ radical via a reactive intermediate or a transition state. A site-specific radical (R^•^) is generated, which then reacts with (TMS)_3_SiH and gives the reduced product (RH), together with “fresh” (TMS)_3_Si^•^ radicals to reinitiate the chain. The chain reactions terminate by radical-radical combination or disproportionation reactions.

(TMS)_3_SiH is an effective reducing agent for the removal of a variety of functional groups. Examples of dehalogenation (Cl, Br and I) and reductive removal of chalcogen groups (SR and SeR) are well known [1,2,3,4,5]. Scheme 2 shows some recent examples of debromination starting from bromide **1** [6], **2** [7] or **3** [8]. The most popular thermal initiator is azobisisobutyronitrile (AIBN), with a half-life of 1 h at 81 °C. Other azo-compounds are used from time to time depending on the reaction conditions. Et_3_B in the presence of very small amounts of oxygen is an excellent initiator for lower temperature reactions (down to −78 °C).

**Scheme 3:**
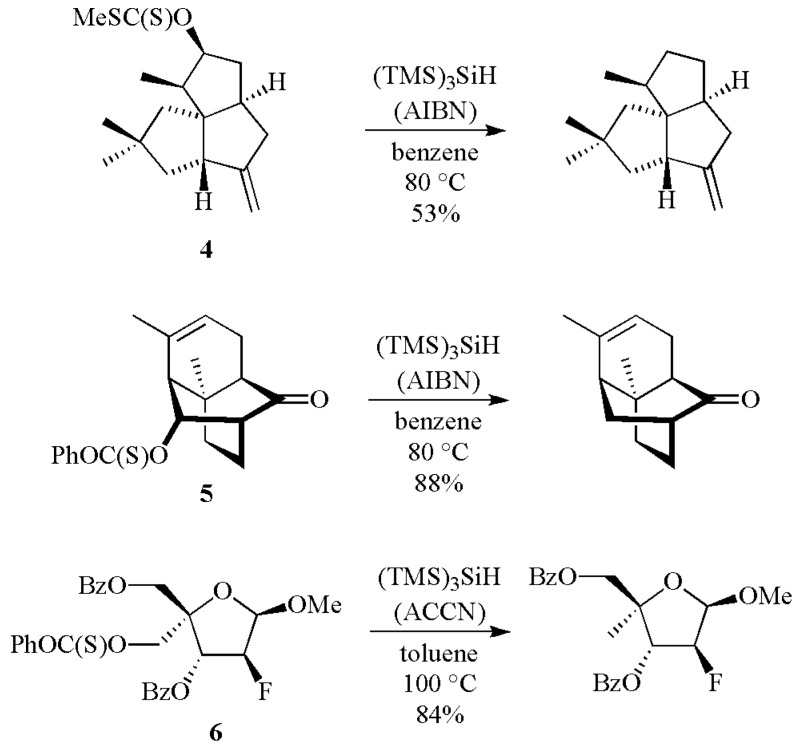
Removal of hydroxy group via thiocarbonyl derivatives by reaction with (TMS)_3_SiH.

For the removal of a hydroxy group, the strategy starts from a thiocarbonyl derivative (e.g., *O*-arylthiocarbonate, *O*-thioxocarbamate, thiocarbonyl imidazole or xanthate) or a selenocarbonate [1,2,3,4,5,9]. Two examples of thiocarbonyl derivative of secondary alcohols are provided with the xanthate **4** [10] and the *O*-phenylthiocarbonate **5** [11] (Scheme 3). The hydroxyl group removal of primary alcohols can be achieved using a thiocarbonyl derivative like the *O*-phenylthiocarbonate derivative **6**, at slightly higher temperature than the deoxygenation of secondary alcohols [12].

**Scheme 4:**
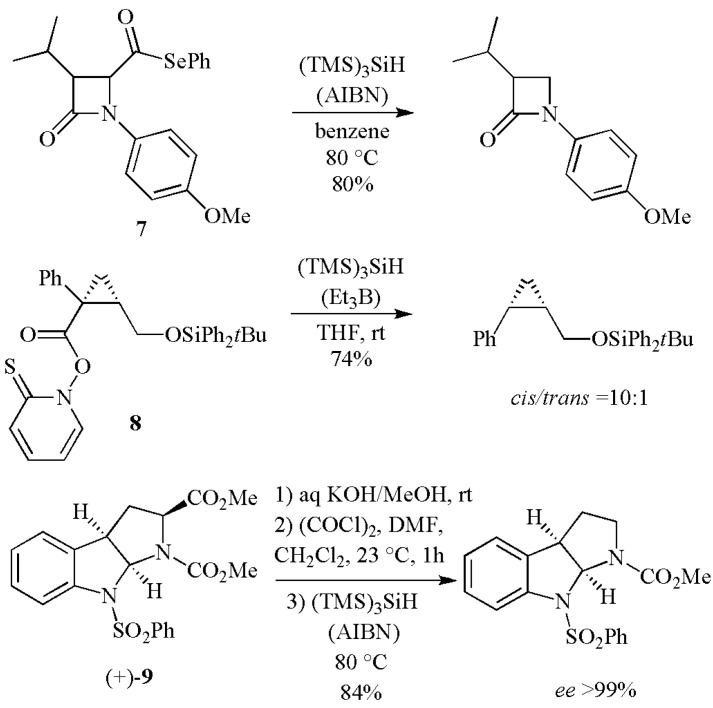
Removal of functional groups by reaction with (TMS)_3_SiH.

A variety of acyl derivatives (such as acyl chlorides, phenylseleno esters or *N*-hydroxypyridine-2-thione esters) can be used for decarbonylation and reductive decarboxylation with (TMS)_3_SiH. Three examples are shown in Scheme 4. The phenylseleno ester **7** afforded the decarbonylated *β*-lactam in good yield [13]. The *N*-hydroxypyridine-2-thione ester **8** was used in the key step to construct the chiral *cis*-cyclopropane structure in compounds designed as antidopaminergic agents [14]. Hydrolysis of the methyl ester followed by decarbonylation at the C2 position of hexahydropyrroloindole (+)-**9** afforded the desired tricyclic product in a 84% yield and >99% *ee* [15].

(TMS)_3_SiH is also useful for the reduction of nitroxides to secondary amines [16] and of phosphine sulfides and phosphine selenides to give the corresponding phosphines [17,18]. Several radical-based reductions with (TMS)_3_SiH have been expanded towards technological applications. Examples are the radical based transformations in a microstructured reaction device, where deoxygenation and dehalogenation reactions are found to be highly efficient [19] or the removal of terminal thiocarbonyl group from polystyrene [20].

**Scheme 5:**
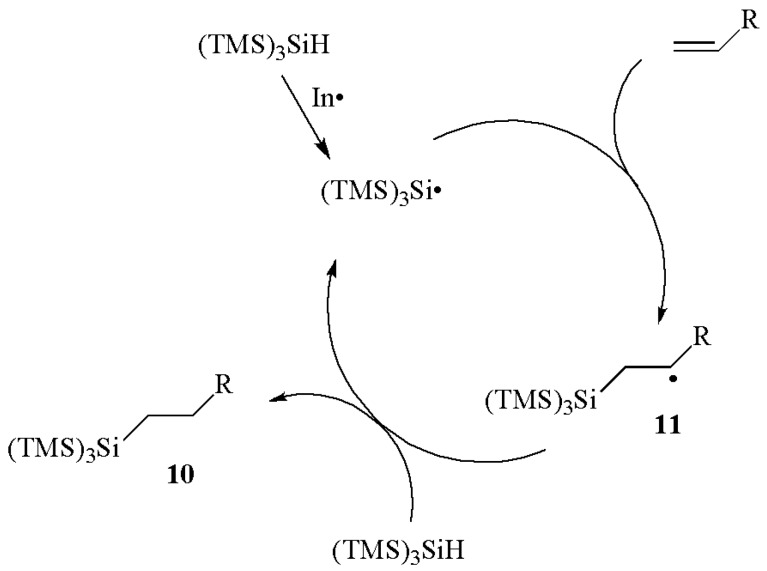
Hydrosilylation of alkenes by (TMS)_3_SiH.

The radical-based hydrosilylation of carbon-carbon double or triple bonds by (TMS)_3_SiH is an important class of reactions [2,3,4,5,21]. These reactions are highly regioselective (*anti*-Markovnikov) and give (TMS)_3_Si-substituted compounds in good yields. Hydrosilylation of monosubstituted olefins is an efficient process in the case of both electron-rich and electron-poor olefins affording silanes **10** (Scheme 5). The initially generated silyl radical adds to the double bond to give a radical adduct **11**, which then reacts with the silicon hydride and gives the addition product, together with “fresh” silyl radicals to continue the chain. Rate constants for the reaction of (TMS)_3_Si^•^ radical with a variety of monosubstituted olefins were measured by laser flash photolysis techniques [22] (see Section 4.1. for more details).

**Scheme 6:**
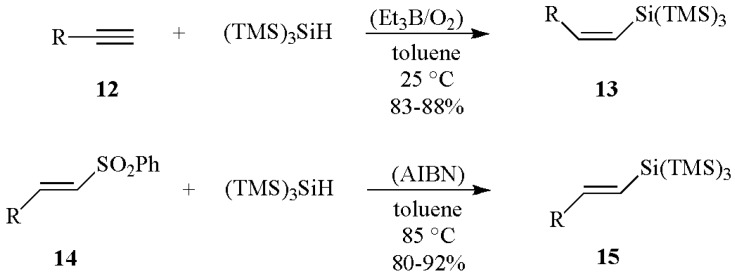
Hydrosilylation of alkynes and silyldesulfonylation by (TMS)_3_SiH.

High *cis* stereoselectivity is observed for the reaction of the alkynes **12** (R = alkyl or aryl) with (TMS)_3_SiH, initiated by Et_3_B/O_2_ at room temperature (Scheme 6). On the other hand, radical-mediated silyldesulfonylation of various (*E*)-vinyl sulfones **14** with (TMS)_3_SiH provides access to (*E*)-vinyl silanes **15 ** (Scheme 6) [23,24]. These highly stereoselective reactions presumably occur via a radical addition followed by *β*-scission with the ejection of PhSO_2_^•^ radical. Hydrogen abstraction from (TMS)_3_SiH by the PhSO_2_^•^ radical completes the cycle of these chain reactions.

The reaction of 2-substituted allyl phenyl sulfides or sulfones with (TMS)_3_SiH give the corresponding 2-substituted allyl tris(trimethylsilyl)silanes in high yields [25], some of which found application as precursor of (TMS)_3_Si^•^ radicals by *β*-fragmentation, and can efficiently mediate group transfer processes [26].

The reaction of (TMS)_3_SiH with oxygen occurs spontaneously and slowly at ambient temperature to form siloxane as the sole product [2,3,4,5] (see Section 4.3. for more details). The rate constant for the spontaneous reaction of (TMS)_3_SiH with molecular oxygen was determined to be ~3.5 × 10^−5^ M^−1^ s^−1^ at 70 °C [27].

**Scheme 7:**
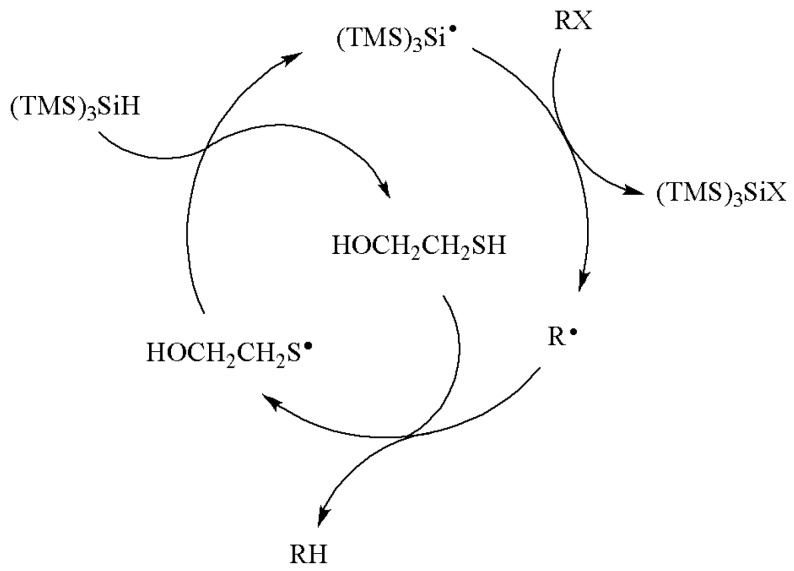
Removal of the X group using the (TMS)_3_SiH/HOCH_2_CH_2_SH reducing system.

The reactivity of (TMS)_3_SiH has also been expanded to fast trapping reducing systems using (TMS)_3_SiH/thiol couple [28], in analogy with polarity-reversal catalysis in the radical-chain reduction introduced by Roberts [29]. The mechanism in Scheme 7 illustrates the propagation steps with 2-mercaptoethanol as the thiol. Its role is to act as the hydrogen donor and then to be regenerated by reaction of thiyl radical with silane. The rate constants of primary alkyl radicals with (TMS)_3_SiH/RSH and (TMS)_3_SiH/ArSH are in the range of 0.9–8 × 10^7^ and 0.75–1.5 × 10^8^ M^−1^ s^−1^, respectively, at 80 °C [28]. Therefore, the role of thiol is to modulate the hydrogen donor ability of the system and allow the fast reaction of carbon-centered radicals with the thiols to be studied.

**Scheme 8:**
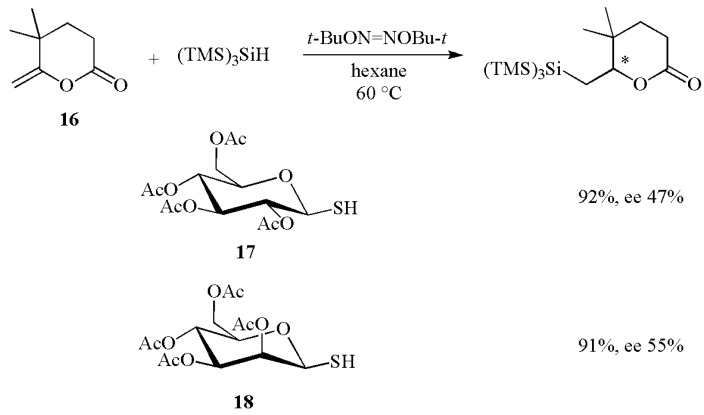
Enantioselective hydrosilylation using the (TMS)_3_SiH/RSH reducing system.

**Scheme 9:**
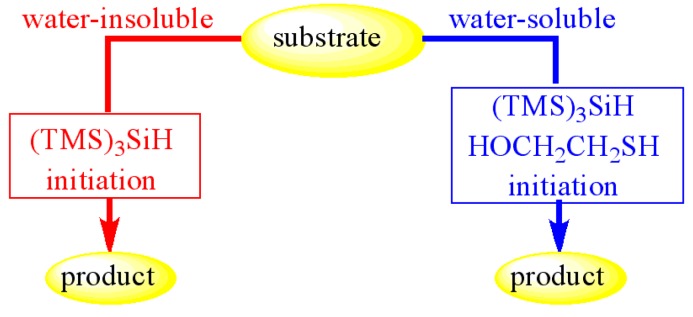
Radical reactions in water using (TMS)_3_SiH or (TMS)_3_SiH/HOCH_2_CH_2_SH.

Thiols have also been shown to catalyze the addition of (TMS)_3_SiH to alkenes. Scheme 8 shows the hydrosilylation of methylenelactone **16** using optically active thiols as catalysts, like the thioglucose tetraacetate **17** or the *β*-mannose thiol **18**, to occur in excellent yields and good enantiomeric purities [30].

(TMS)_3_SiH is not soluble in water and does not significantly react with water at 100 °C for several hours, motivating interest in applying it to radical reactions in water [31,32]. Water-insoluble substrates, suspended with (TMS)_3_SiH and the radical initiator ACCN (1,1′-azobis-(cyclohexanecarbonitrile)), in aqueous medium at 100 °C under vigorous stirring, can be reduced in good yields. This procedure does not work with water-soluble substrates, however when a catalytic amount of the amphiphilic 2-mercaptoethanol is coupled to (TMS)_3_SiH, it becomes a very efficient system for the reduction of different organohalides (Scheme 9).

**Scheme 10:**
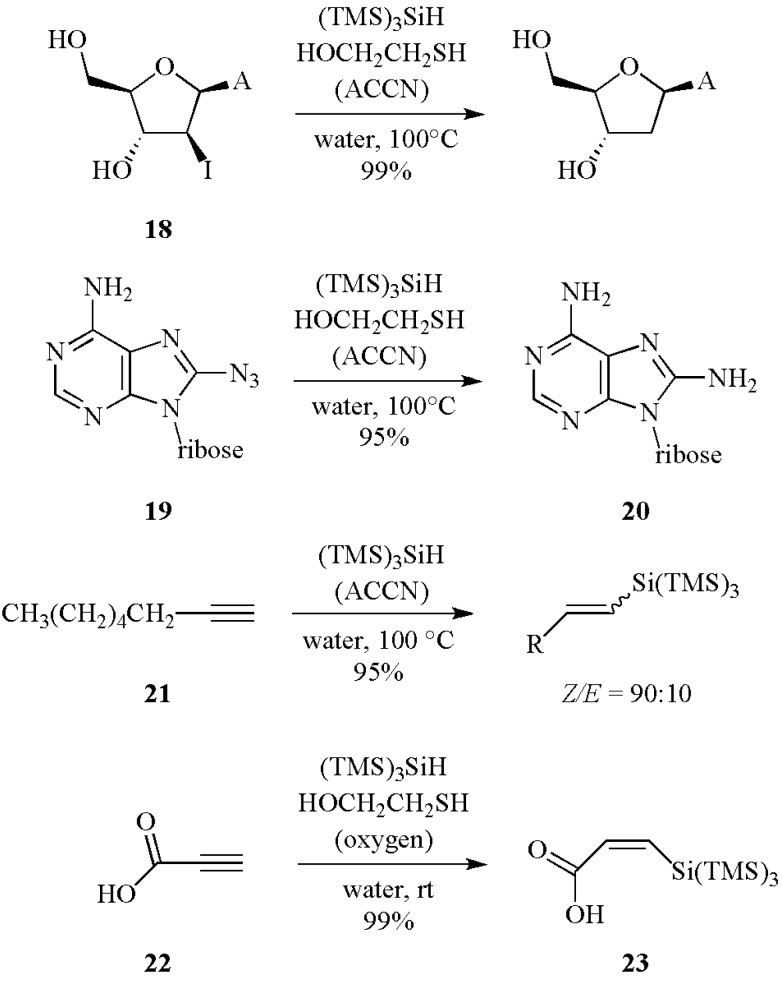
Reduction and hydrosilylation reactions in water using (TMS)_3_SiH.

Two examples of water soluble nucleosides are shown in Scheme 10, with the dehalogenation of iodide **18**, and the reduction of 8-azidoadenosine **19** that affords the corresponding amino derivative **20** [31]. The water insoluble alkyne **21** afforded the hydrosilylation product in good yield and in *Z/E* ratio of 90:10. Under dioxygen initiation at room temperature the same reaction product is obtained in similar yield but in a *Z/E* > 99:1. On the other hand, the water soluble alkyne **22** afforded in quantitative yield the isomer **23**, either under dioxygen initiation at room temperature or with ACCN at 100 °C [32].

## 3. (TMS)_3_SiH as Mediator of Consecutive Radical Reactions

**Scheme 11:**
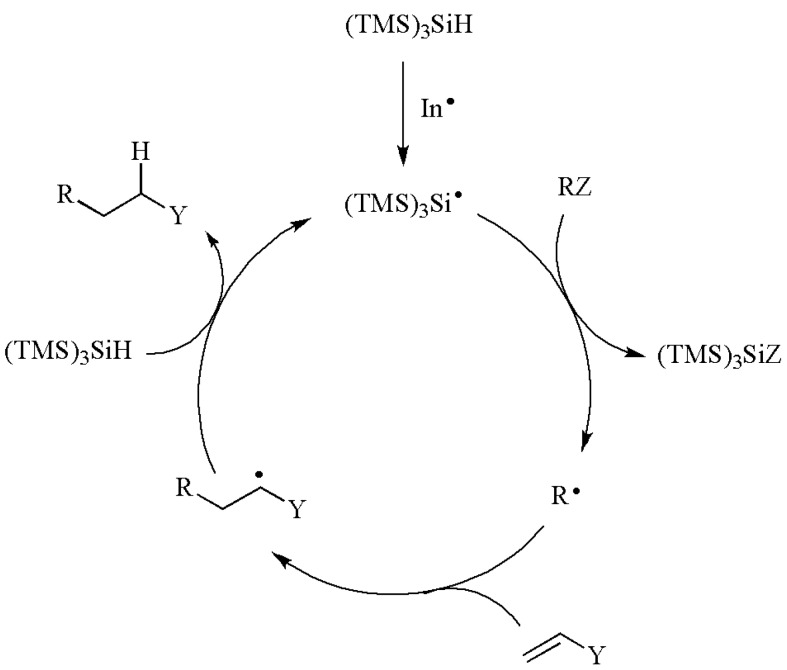
Reductive alkylation of alkenes by (TMS)_3_SiH.

The carbon-centered radical, resulting from the initial atom or group removal (Scheme 1) or by addition to an unsaturated bond (Scheme 5), can be designed to undergo a number of consecutive reactions prior to the H-atom transfer. The key step in these consecutive reactions generally involves the intra- or inter-molecular addition of the carbon-centered radical to a multiple-bonded carbon acceptor. As an example, the propagation steps for the reductive alkylation of alkenes by (TMS)_3_SiH are shown in Scheme 11. This sequence requires a radical reagent, which is fast to abstract the group Z from the starting material, but not too fast to reduce the corresponding R^•^ prior its addition to the multiple bonds.

(TMS)_3_SiH as mediator has contributed substantially in the area of multi-step radical reactions giving the best results, compared to other reducing reagents. For example, Nicolaou and co-workers found (TMS)_3_SiH to be a superior reagent in the radical-based approach toward the synthesis of azadirachtin, an antifeedant agent used as insecticide, as well as in other related systems [33,34].

**Scheme 12:**
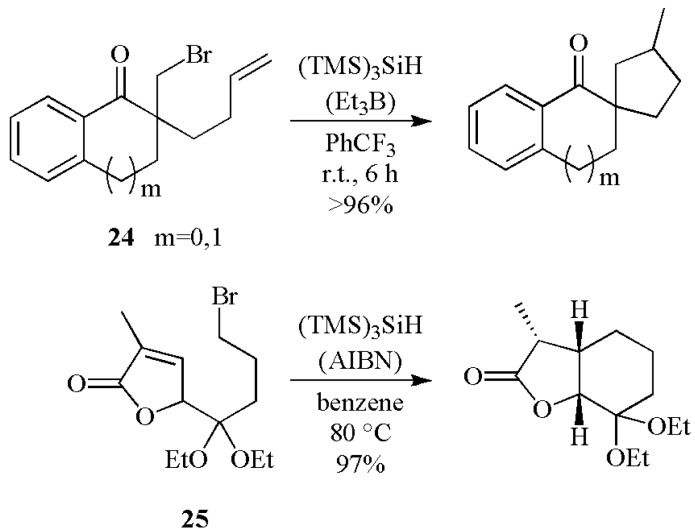
Carbocycles construction using (TMS)_3_SiH.

Scheme 12 shows the synthesis of carbocycles from the recent work in the area of intramolecular reactions. The 5-membered ring formation has been used for preparing spiro-compounds starting from bromide **24** in excellent yields [35], whereas bromide **25** is the starting material of 6-membered radical cyclization affording the complete diastereocontrol of three contiguous stereocenters [36].

**Scheme 13:**
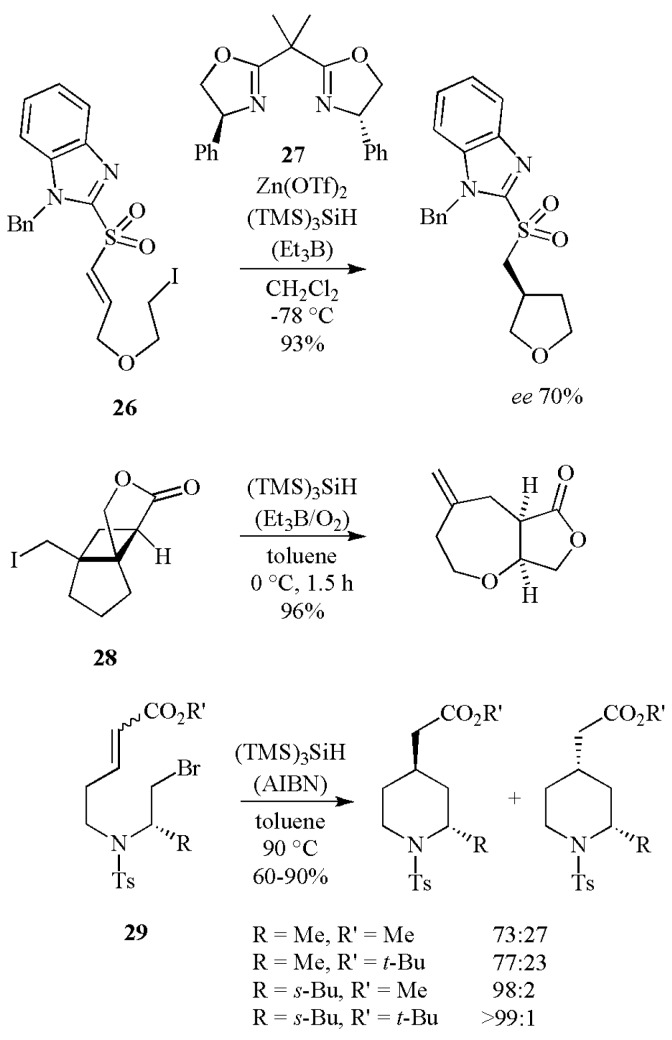
Construction of furan-ring and cyclic amines using (TMS)_3_SiH.

Scheme 13 shows the synthesis of oxygen- and nitrogen-containing heterocycles from the recent work in the area of intramolecular reactions. Enantioselective radical cyclizations have been performed by using chiral Lewis acids together with (TMS)_3_SiH as reducing agent. An example with a 70% *ee* is given, in which the selective coordination of one of the enantiotopic sulfonyl oxygen atoms in *ω*-iodoalkenyl sulfone **26** is achieved using Zn(OTf)_2_-bis(oxazoline)-Ph **27** [37]. The (iodomethyl)-cyclobutane derivative **28** undergoes ring expansion with (TMS)_3_SiH to afford the *cis*-oxepane in an excellent yield (96%) [38]. A diastereoselective radical route to 2,4-disubstituted piperidines has been achieved in good yields (60–90%) by cyclization of 7-substituted-6-aza-8-bromooct-2-enoates (**29**) using (TMS)_3_SiH as the reducing agent [39]. Four examples are given, where the *trans/cis* diastereomeric ratios range from 73:27 for R = R´ = Me (90% yield) up to >99:1 for R = *s*-Bu and R′ = *t*-Bu (60% yield). Although the bulkiness of the ester does not appear to have a significant effect on the stereoselectivity, the bulkiness of the 2-substituent increases the pseudo A^1,3^ strain and favors the *trans* product.

**Scheme 14:**
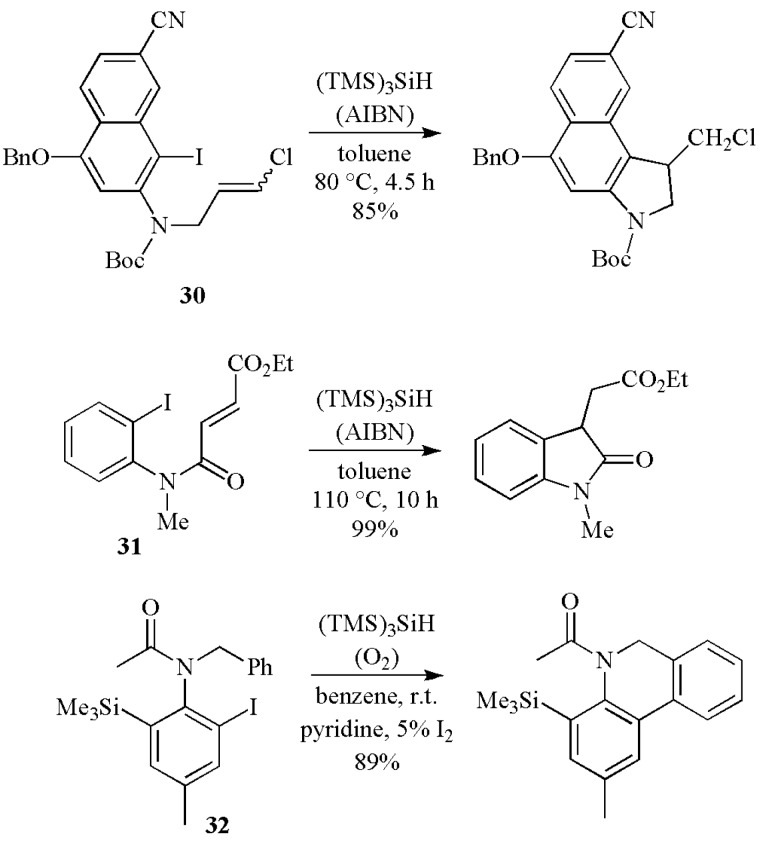
Construction of nitrogen heterocycles using (TMS)_3_SiH.

The aryl radical cyclization has been successfully used for the preparation of a variety of derivatives (Scheme 14). Radical cyclization of **30** mediated by (TMS)_3_SiH under standard reaction conditions worked well to afford the cyclic product in a 85% yield [40]. The second example is shown in the cyclization of **31** mediated by (TMS)_3_SiH and AIBN in refluxing toluene [41]. The (TMS)_3_SiH mediated cyclization of aryl iodide **32** is facilitated by oxidative rearomatization with oxygen [42]. Actually AIBN is not necessary for the good performance of the reaction. 

**Scheme 15:**
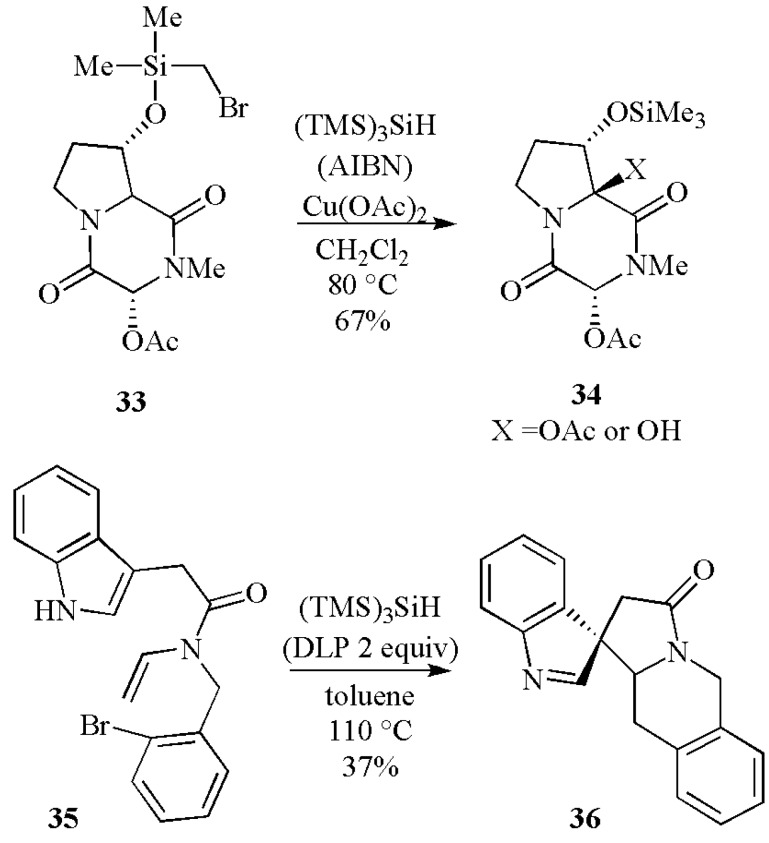
Examples of unimolecular radical processes followed by oxidation.

Interesting examples of unimolecular radical reactions followed by oxidation are illustrated in Scheme 15. A radical-promoted functionalization of the angular carbon was developed with the readily available bromomethylsilyl acetal **33** [43]. Large excess of Cu(OAc)_2_ (10 equiv) and AIBN (3 equiv) were required together with (TMS)_3_SiH (3 equiv). The electron-rich captodative radical produced after the 1,5-hydrogen atom transfer was readily oxidized by Cu(OAc)_2_ to afford the desired acetate **34** in a 42% yield, together with the corresponding alcohol in 25% yield. On the other hand, when bromide **35** is treated with (TMS)_3_SiH and 2 equiv. of dilauroyl peroxide (DLP), the spiro-derivative **36** was obtained in one-pot and a 37% yield. The organic peroxide appears to act as the initiator and the oxidant. In this case the 6-*endo* cyclization of aryl radical onto an enamide double bond is followed by a consecutive oxidative-ionic spirocyclization at C-3 of an indole nucleus [44].

**Scheme 16:**
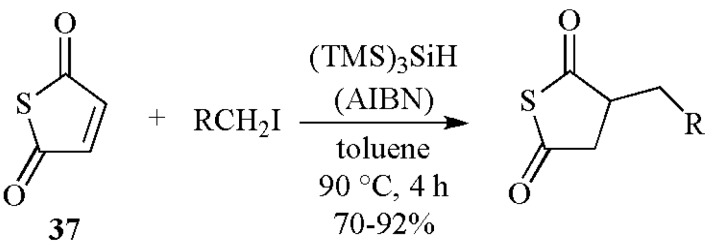
Examples of intermolecular carbon-carbon bond formation.

The intermolecular C–C bond formation mediated by (TMS)_3_SiH is illustrated in Scheme 11 has been the subject of several synthetically useful investigations. A recent example is shown in Scheme 16, where a variety of iodoalkyl derivatives undergo clean free-radical addition to thiomaleic anhydride **37** to give substituted thiosuccinic anhydrides in high yield on treatment with tris(trimethylsilyl)silane and a radical initiator [45].

**Scheme 17:**
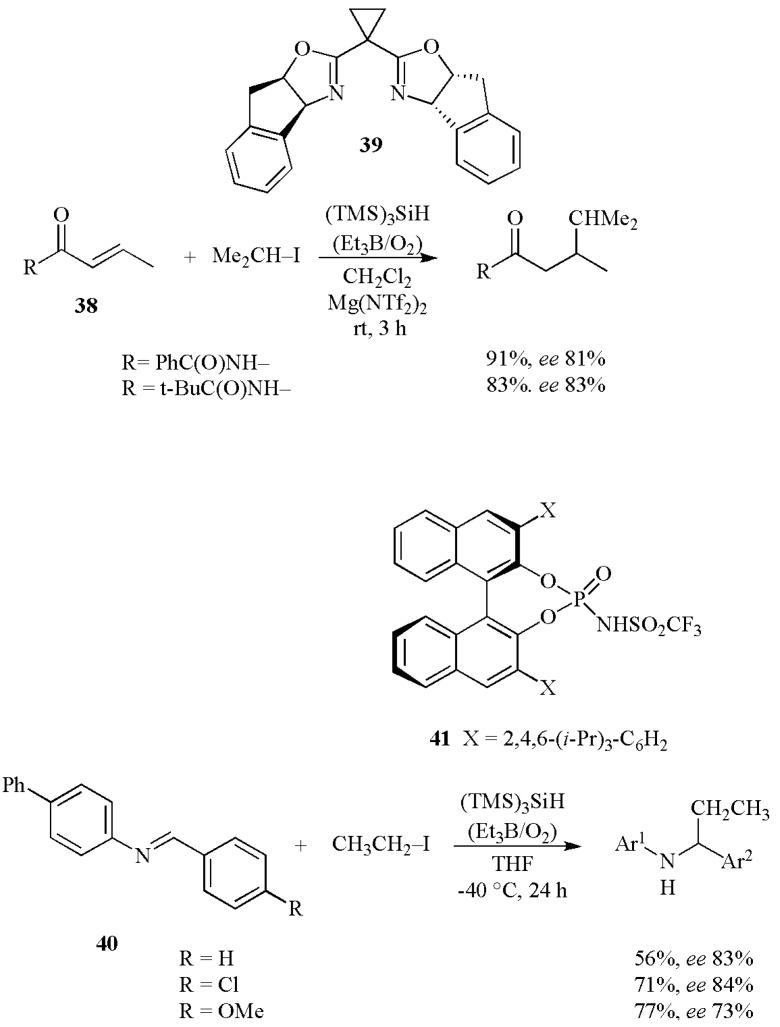
Enantioselective radical reactions using (TMS)_3_SiH as mediator.

Scheme 17 shows two classes of intermolecular C–C bond formation carried out by Lewis acid mediated enantioselective radical reactions [46,47]. Conjugated radical additions to a variety of *α,β*-unsaturated imides **38** were performed at room temperature using 3 equiv of the hydrogen donor (TMS)_3_SiH and Et_3_B/O_2_ as initiator in CH_2_Cl_2_ [46]. The chiral Lewis acid derivative from magnesium triflimide and bisoxazoline **39** (30 mol%) was used. Phenyl and *tert*-butyl imide substituents provided enantioselectivity of 81% and 83%, respectively. On the other hand, binaphthol-derived chiral phosphoric acid catalysts **41** (0.3 equiv) were applied to radical addition reactions of imines **40** and provided chiral amines in good yields and good enantioselectivities [47]. The enantioselectivities were not affected by the electronic properties of imines.

**Scheme 18:**
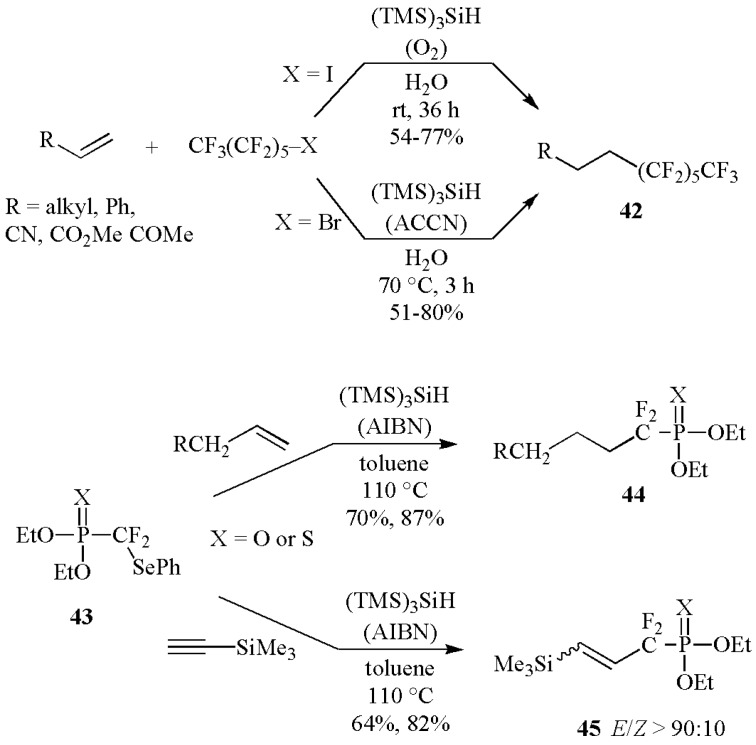
Fluoroalkyl radicals addition to C–C multiple bonds mediated by (TMS)_3_SiH.

The (TMS)_3_SiH-mediated addition of fluoroalkyl radicals to alkenes has also been reported [48,49]. The addition of perfluoroalkyl iodides or bromides to a variety of alkenes afforded the expected adducts **42** with moderate to good yields in water as the solvent (Scheme 18) [48]. Two versatile and convenient methodologies were individuated depending on the nature of the starting materials, *i.e.*, with iodides the initiation step with molecular oxygen is enough to ensure smooth reaction at room temperature, whereas with bromides the azo-initiation at 70 °C was necessary. Phenylseleno derivatives **43** are found to be good precursors of phosphonodifluoromethyl and phosphonothiodifluoromethyl radicals under normal reaction conditions, *i.e.*, refluxing toluene and AIBN as initiator (Scheme 18). Moreover, when generated by the (TMS)_3_Si^•^ attack on **43** and in the presence of alkenes or alkynes, they gave access to *α*,*α*-difluorinated derivatives **44** and *β*,*γ*-unsaturated adducts **45**, via carbon-carbon bond formation. The phosphonothio derivatives are obtained in higher yields than phosphonates in both reactions [49].

The increasing popularity of radical reactions is certainly due to the so-called “tandem or cascade reaction”, *i.e.*, the ability of forming and breaking several bonds in one-pot procedure. For example, substituted 5′,8-cyclopurine and 5′,6-cyclopyrimidine nucleosides have been synthesized by combination of radical translocation/cyclization steps (Scheme 20) [50,51]. Treatment of the 8-bromo derivative **46** or 6-phenylseleno derivative **48** with (TMS)_3_SiH and AIBN in benzene at 80 °C for 4 h resulted in the cyclonucleosides **47** and **49**, respectively. The initially generated radical on the base moiety by Br or PhSe abstraction translocates to C5′ position of sugar by hydrogen atom transfer followed by cyclization on the base moieties. Rearomatization or hydrogen abstraction completes the cycle affording the cyclonucleosides in good yields and diastereomeric ratio in favor of (5′*S*)-isomer. The most interesting example is the synthesis of (5′*S*)- and (5′*R*)- 5′,8-cyclo-2′-deoxyadenosine (**47**), a tandem-type lesions observed among the DNA modifications [52].

**Scheme 19:**
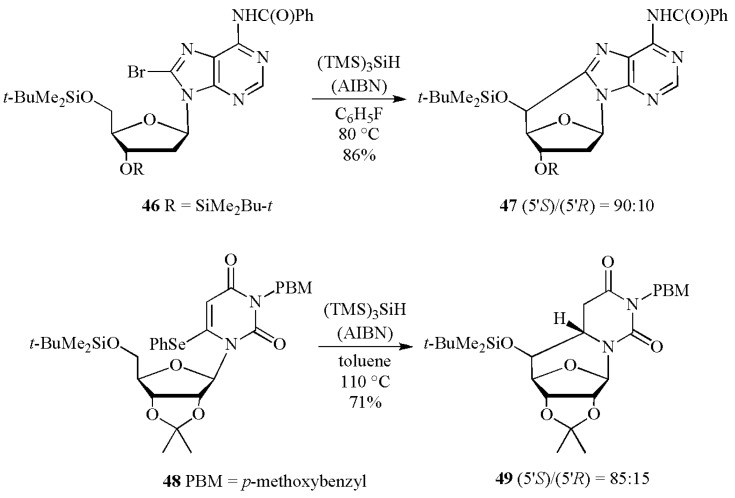
Synthesis of cyclonucleosides mediated by (TMS)_3_SiH.

**Scheme 20:**
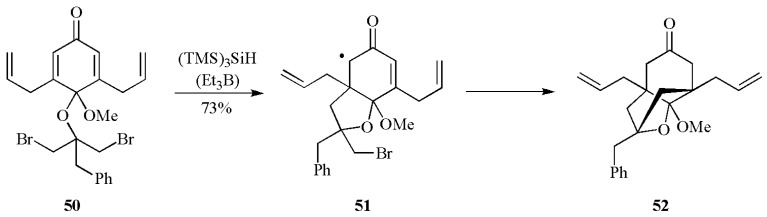
Two consecutive reductive cyclizations mediated by (TMS)_3_SiH.

A nice example of two consecutive 5-*exo* cyclizations in a one-pot reaction is illustrated in Scheme 20. Starting from dibromide **50**, the first cyclization via the radical **51** affords the desymmetrization of the molecule, whereas the second cyclization provides the bicyclic core **52** that is common of guttiferone family of natural products [53].

**Scheme 21:**
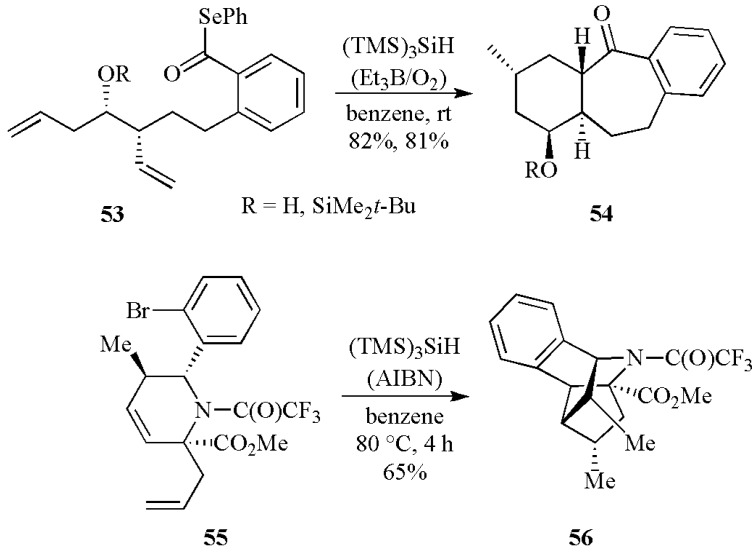
Tandem cyclizations mediated by (TMS)_3_SiH.

Radical-based tandem cyclizations have been successfully applied in various systems. Reactions that feature a 7-*exo* acyl radical cyclization followed by a 6-*exo* or 5-*exo* alkyl radical cyclization proceed with very good yields and diastereoselectivities [54]. As an example the treatment of **53** with (TMS)_3_SiH at room temperature provided the tricycles **54** in excellent yields as single diastereomer. Interestingly, the bulky silyl ether moiety is not required to achieve stereoselectivity in this process. Radical cyclizations of tetrahydropyridine scaffolds have been used to access diverse skeletal frameworks [55]. Treatment of the allylated tetrahydropyridine **55** with (TMS)_3_SiH and AIBN in refluxing benzene resulted in a single diastereomer **56** in a 65% isolated yield (Scheme 21).

**Scheme 22:**
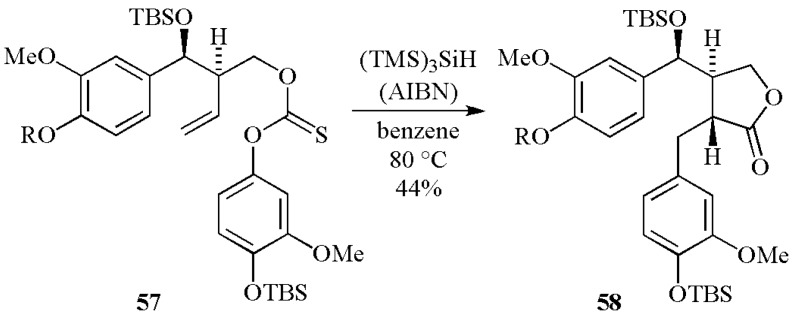
Domino reactions mediated by (TMS)_3_SiH.

A radical carboxyarylation approach is also introduced as the key step in the total synthesis of several biologically important natural products [56,57]. Treatment of thiocarbonate derivatives **57** (R = Me or TBS) with 1.1 equiv of (TMS)_3_SiH in refluxing benzene and in the presence of AIBN (0.4 equiv. added over 6 h) as radical initiator, produced compound **58** in a 44% yield (Scheme 22). This remarkable transformation resulted from a radical cascade, involving (TMS)_3_Si^•^ radical addition to a thiocarbonyl function, 5-*exo* cyclization and intramolecular 1,5-*ipso* substitution with the final ejection of (TMS)_3_SiS^•^ radical.

**Scheme 23:**
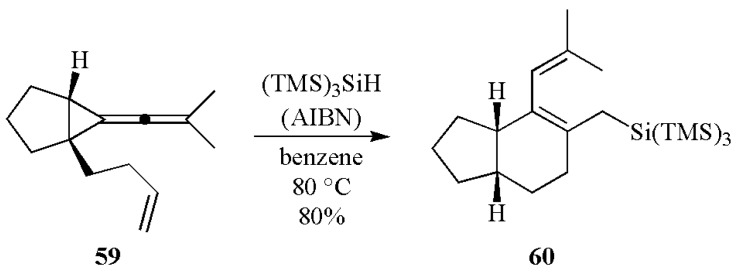
Radical cascade process mediated by (TMS)_3_SiH.

Another case of radical cascade processes is illustrated in Scheme 23. Treatment of the butenyl-substituted allenecyclopropane **59** with (TMS)_3_SiH/AIBN resulted in facile radical cyclizations into the cyclopropane-carbon centres of the allene moieties, followed by cyclopropane ring-opening and allene isomerisation, leading to the bicyclic 1,3-diene **60** [58].

## 4. Applications of (TMS)_3_SiH in Photopolymerization Reactions

The search for new photoinitiated systems remains important. Particularly, the design of new sources of radicals is the subject of numerous research efforts. Indeed, new initiating radicals with different properties and/or enhanced reactivity should provide new opportunities for the development of photocurable materials [59,60,61].

Silyl radicals are already characterized by a widespread use in hydrosilylation and reduction reactions in organic chemistry (see previous sections). However, quite surprisingly these reactive species were not really employed in the polymer science area. It has been found that silyl radicals, in particular the (TMS)_3_Si^•^, were characterized by interesting properties for applications in polymerization initiating systems: (i) a high inherent reactivity of the silyl radicals for the addition to double bonds which can be exploited in Free Radical Polymerization (FRP) and (ii) a low ionization potential (IP) which is associated with a favorable oxidation process and the formation of silylium cations [22,62,63]. These latter structures can be very attractive to initiate the ring opening polymerization process (e.g., polymerization of epoxides). This process is called Free Radical Promoted Cationic Polymerization (FRPCP): the initiating cations are generated from oxidation of free radicals [64,65,66,67].

Based on this outstanding reactivity, new initiating systems incorporating the (TMS)_3_SiH were proposed recently [62,63,68,69,70,71,72,73,74,75,76,77,78]. In the present review, some examples of newly developed photoinitiated systems based on this latter compound are presented. They exhibit a high reactivity both in FRP [77] and FRPCP [78] under air. Some specific applications are provided. The high reactivity of (TMS)_3_SiH for the conversion of peroxyls to hydroperoxides [79,80], as well as the ability of (TMS)_3_Si^•^ to efficiently consume O_2_ [27,81], are also found highly interesting to overcome the classical and well known oxygen inhibition of the FRP processes.

### 4.1. Formation and Reactivity of Silyl Radicals in Photoinitiated Systems

A large number of rate constants for the reactions of (TMS)_3_Si^•^ and other silyl radical reactions are known [3,22,82]. For the production of a directly observable silyl radical, a classical way consists of the interaction of photogenerated *tert*-butoxyl radical (*t*-BuO^•^) or benzophenone triplet state (^3^BP) with the corresponding silane [22,83]. The interaction of the silane with a ketone triplet state (such as in benzophenone (BP) or thioxanthone (TX) derivatives) leads to hydrogen abstraction which generates a ketyl (BPH^•^ or TXH^•^) and (TMS)_3_Si^•^ radical. The ketyl or silyl radical quantum yields are close to 1 for (TMS)_3_SiH [22]. In Table 1, the rate constants for the reactions (1) and (2) are compared to the kinetic data obtained for triethylsilane. The values are about ten times higher for (TMS)_3_SiH compared to Et_3_SiH. This highlights the role of the Si—H bond dissociation enthalpy, being 353.5 and 398 kJ mol^–1^ for (TMS)_3_SiH and Et_3_SiH, respectively [82,85]. 

**Table 1:** Rate constants for the hydrogen abstraction from silanes by ^3^BP and *t-*BuO^•^ at 25 °C (*Φ*, ketyl radical quantum yields).

	*k*_H_(^3^BP), 10^7^ M^−1^ s^−1^	*k*_H_(*t-*BuO^•^), 10^7^ M^−1^ s^−1^
Et_3_Si—H	0.83 ^a^; 0.96 ^b^ (*Φ* = 0.81)	1.0 ^a^; 0.57 ^b^
(TMS)_3_Si—H	10.2 ^a^ (*Φ* = 0.95)	8.5 ^a^; 11.0 ^c^

^a^ From reference [22]; ^b^ From reference [83]; ^c^ From reference [84].


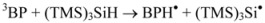
(1)


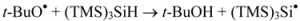
(2)

(TMS)_3_Si^•^ reacts with monomer (M) double bond through an addition process (3). This process is efficient and can be followed by the subsequent addition of monomer units corresponding to a propagation reaction (4).


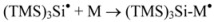
(3)


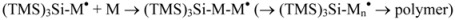
(4)

The rate constants for the addition (*k*_add_) of (TMS)_3_Si^•^ and Et_3_Si^•^ to a large range of alkenes are summarized in Table 2 [22,86,87]. Both electron rich (vinyl acetate, vinyl ether) and electron poor alkenes (acrylonitrile, acrylate) were investigated. Interestingly for (TMS)_3_Si^•^, the striking feature is that the *k*_add_ values remain very high for all the investigated alkenes. Therefore, (TMS)_3_Si^•^ exhibits a rather low selectivity toward substrates ranging from electron poor to electron rich alkenes [22]. Based on quantum mechanical calculations, this low selectivity for (TMS)_3_Si^•^ was explained by antagonist polar and enthalpy effects [22]. For Et_3_Si^•^ the polar effects are not really important and the selectivity is much higher. Rate constants (*k*_add_) decreases by a factor of only 200, going from styrene to vinyl ether, for (TMS)_3_Si^•^ but this factor is 2500 for Et_3_Si^•^. Under the same conditions, for carbon centered radicals, a factor higher than 1000 is usually observed [88].

**Table 2:** Rate constants (*k*_add_) for the addition of (TMS)_3_Si^•^ and Et_3_Si^•^ radicals to different alkenes.

*k*_add _(M^−1^ s^−1^)	Styrene	Acrylonitrile	Methyl acrylate	Vinyl acetate	Vinyl ether	Ref.
(TMS)_3_Si^•^	5.1 × 10^7^	5.1 × 10^7^	2.2 × 10^7^	1.2 × 10^6^	2.1 × 10^5^	[22]
	5.9 × 10^7^	6.3 × 10^7^	9.7 × 10^7 a^			[86]
Et_3_Si^•^	2.1 × 10^8^	1.1 × 10^9^	2.4 × 10^8^	3.5 × 10^6^	9 × 10^4^	[22]
	2.2 × 10^8^	1.1 × 10^9^				[87]

^a ^Ethyl acrylate.

For the initiation of polymerization reactions (e.g., addition onto acrylate), (TMS)_3_Si^•^ is much better than the aminoalkyl radical derived from a very well-known reference co-initiator (ethyldimethyl-aminobenzoate EDB) (*k*_add _~ 5 × 10^5^ M^−1^ s^−1^) [89]. These data highlight the high potential of initiating systems based on (TMS)_3_Si^•^ radical.

### 4.2. (TMS)_3_Si^•^ as a Initiator for Free Radical Polymerization (FRP)

Mainly two different photoinitiated systems exist: type I and type II. For type I photoinitiated systems, the free radicals are generated by a homolytic cleavage process under light irradiation. For type II systems, free radicals are generated by a hydrogen abstraction reaction between a photoinitiator excited state (e.g., triplet state of ketone) and a hydrogen donor (called a co-initiator) [59,60,61].

**Figure 1:**
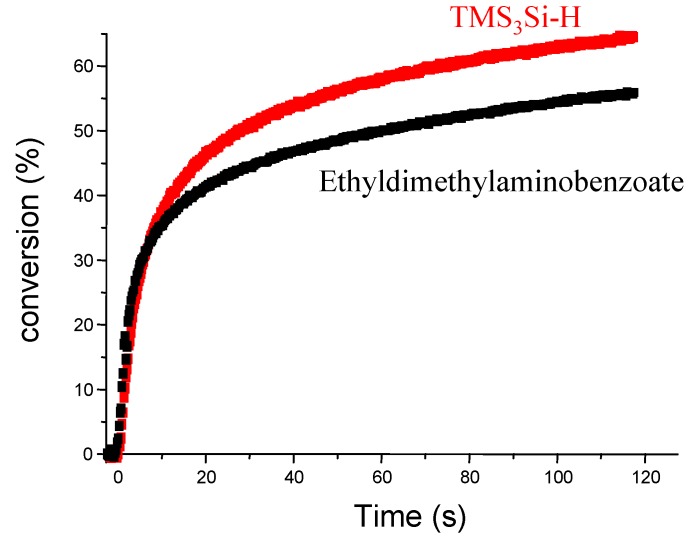
Polymerization profiles of trimethylolpropane triacrylate. Benzophenone/ (TMS)_3_SiH (1%/2%) *vs.* benzophenone/ethyldimethylaminobenzoate (1%/2%) in laminate.

Recently, a large series of silanes (R_3_SiH) has been investigated as new co-initiators for FRP in type II systems [63]. A hydrogen abstraction between the photoinitiator excited state and the R_3_SiH, reaction (1), corresponds to the pivotal process of the initiation mechanism. The quantum yields in silyl radicals must be very high and ideally close to 1 like in (TMS)_3_SiH (Table 1). The high reactivity of silyl radicals for the initiation process ensures a good efficiency for these photoinitiated systems. Different photoinitiators were recently proposed in combination with (TMS)_3_SiH to extend the spectral sensitivity of these initiating systems, *i.e.*, benzophenone, 2-isopropylthioxanthone, camphorquinone, Eosin-Y and thiopyrylium salts. Actually, the proposed systems can efficiently cover the spectral range from 300 nm to about 600 nm [77]. Systems for red light based on violanthrone photosensitizers were recently proposed with an increased spectral sensitivity (up to 700 nm) [90]. Interestingly, the polymerization profiles for acrylate matrix were found better than those obtained in the presence of a reference amine co-initiator (ethyldimethylaminobenzoate-EDB) (Figure 1) with an increase of both the polymerization rates and the final conversions. The ability of (TMS)_3_SiH in the initiation process under air was excellent (better than amines) and the oxygen inhibition usually encountered in FRP was reduced. This behavior will be discussed below for the ability of (TMS)_3_SiH to overcome the oxygen inhibition for the FRP process [91].

### 4.3. (TMS)_3_SiH: A New Additive to Overcome the Oxygen Inhibition in FRP Process

The oxygen inhibition for the FRP process is usually associated with the conversion of initiating or propagating radicals to stable peroxyls by reaction with O_2_. A drastic decrease of both polymerization rate and final conversion is found for the FRP process under air. (TMS)_3_SiH was found to be the best silane to overcome this oxygen detrimental effect in many FRP processes. A detailed mechanistic investigation revealed that different processes are important for this specific behavior: 

(i) (TMS)_3_Si^•^ radicals are efficient to trap oxygen (5) with an interaction rate constant close to 2 × 10^9^ M^−1^ s^−1^ [27,91]: 


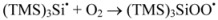
(5)

(ii) Peroxyl radicals are readily trapped by (TMS)_3_SiH [79,80]. The reaction (6) converts stable peroxyls to new initiating (TMS)_3_Si^•^ radicals: this is highly worthwhile to overcome the oxygen inhibition. R can be an initiating or a propagating radical:


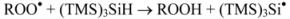
(6)

(iii) Another very important process for the reactivity of (TMS)_3_Si^•^ under air is the fast rearrangement of the associated peroxyl reaction (7) that again re-generates an initiating silyl radical [27,81,91]. The presence of oxygen substitution for the final silyl radical does not decrease the reactivity of this species for the addition onto acrylate e.g., the addition rate constant of the triethoxysilyl radical to methyl acrylate was measured as 5.5 × 10^8^ M^−1^ s^−1^ [63]: 


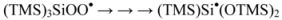
(7)

(iv) Under a polychromatic irradiation, the photodecomposition of the hydroperoxides formed under air can also be expected (reaction (8)). The silyloxyl or alkoxyl radicals can probably easily abstract a hydrogen atom from (TMS)_3_SiH leading to (TMS)_3_Si^•^, or can also initiate the polymerization process:


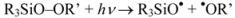
(8)

**Figure 2:**
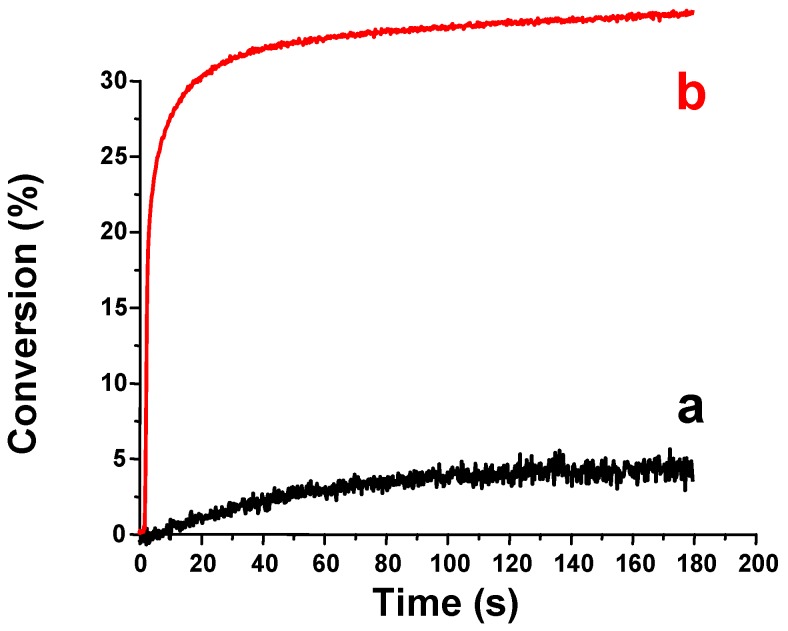
Polymerization profiles of trimethylolpropane triacrylate. Photoinitiated system: (a) diphenyl (2,4,6-trimethylbenzoyl)-phosphine oxide (1% w/w) and (b) diphenyl (2,4,6-trimethylbenzoyl)-phosphine oxide/(TMS)_3_SiH (1%/2% w/w) under air.

All these processes explain quite well the excellent initiating ability found for type II systems based on (TMS)_3_SiH. It can also be used as additive in type I systems for its ability to convert stable peroxyls to new silyl moieties as initiating species (Figure 2: curve (a) absence of (TMS)_3_SiH *vs.* curve (b) presence of (TMS)_3_SiH). For all the investigated systems the presence of (TMS)_3_SiH drastically increases the polymerization rate and the final conversion [62,63,77].

### 4.4. Silyl Radicals in Free Radical Promoted Cationic Polymerization (FRPCP)

The development of ring opening polymerization reactions under conventional irradiation system (λ > 300 nm) is actually rather limited, *i.e.*, this is ascribed to the very low absorption of the aryl iodonium salts IS^+^ used as photoinitiators for these polymerization processes [59,60,61].

For an access to a convenient spectral range corresponding to classical or industrial lamps (λ > 300 nm), a sensitization procedure is required. Among the different approaches developed, the Free Radical Promoted Cationic Photopolymerization FRPCP is actually recognized as an interesting alternative for the use of longer wavelength [64,65,66,67]. FRPCP corresponds to the excitation of a photosensitive system (radical initiator) where a produced radical R^•^ can be oxidized by diphenyliodonium salt, Ph_2_I^+^ (9). The resulting cation R^+^ is the initiating structure for the ring opening polymerization:


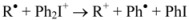
(9)

The search for new radical sources for the FRPCP process still remains important. The free radicals involved must be characterized by (i) excellent oxidation properties for an efficient cation formation process, and (ii) the resulting cation R^+^ must initiate the ring opening polymerization (e.g., the ring opening of epoxides). 

It was previously envisaged in the literature that silyl radicals are characterized by low ionization potentials IP [3,82]. Based on this seminal finding, new FRPCP systems based on silyl radical chemistry were proposed recently [62]. These photosensitizer(PS)/silane/IS^+^ photoinitiated systems were sensitive upon visible light exposure and also under air (especially using diphenyl iodonium hexafluorophosphate (Ph_2_I^+^) as IS^+^). From all the investigated R_3_SiH, (TMS)_3_SiH leads to the best polymerization profiles. These systems are very efficient for the polymerization of epoxy monomers (3,4-epoxycyclohexane)methyl 3,4-epoxycyclohexylcarboxylate; Bis[2-(3,4-epoxycyclohexyl)ethyl]-tetramethyldisiloxane; epoxidized soybean oil (ESO) and limonene dioxide (LDO)) or vinylether monomers (triethylene glycol divinyl ether – DVE-3) or ε-caprolactone [78].

The general mechanism is given in reactions (10)–(13). The *PS/(TMS)_3_SiH interaction yields (TMS)_3_Si^•^ (11). Due to the low silyl IP, a high oxidation rate constant (*k*_ox_) of (TMS)_3_Si^•^ by IS^+^ was measured (12) by laser flash photolysis: *k*_ox_ = 2.6 × 10^6^ M^−1^s^−1^ [62]. The concomitant reduction of Ph_2_I^+^ leads to Ph^•^ that can be easily evidenced by the ESR-spin trapping technique. These phenyl radicals can abstract hydrogen atom from (TMS)_3_SiH with a rate constant of 3 × 10^8^ M^−1^s^−1^ [85] regenerating silyl radicals (13). This latter reaction explains the high Si–H conversion observed in the associated photoinitating systems:


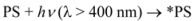
(10)


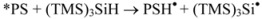
(11)


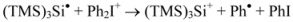
(12)


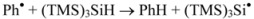
(13)

A large range of photosensitizers including camphorquinone, benzophenone, 2-isopropylthioxanthone, benzyl, Eosin-Y, thiopyrylium salts, acridine diones, coumarin, ketocoumarin, methylene blue, decatungstate, violanthrone derivatives, Iridium or Ruthenium complexes (see also Section 4.5.) were proposed allowing an excellent covering of the 300‑750 nm spectral range for the three-component systems: PS/(TMS)_3_Si-H/Ph_2_I^+ ^[78].

Type I photoinitiators (PI) can also be used in combination with (TMS)_3_SiH for FRPCP [78]. In this latter approach, (TMS)_3_Si^•^ are generated by a hydrogen abstraction reaction between the radicals generated from the PI and the silane, reactions (14) and (15). Reactions (12) and (13) are still operativ: 


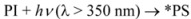
(14)


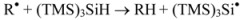
(15)

A large variety of PIs was found to be very efficient for these three-component systems PI/(TMS)_3_SiH/Ph_2_I^+^: phosphine oxide, phenyl bis(2,4,6-trimethyl benzoyl) (BAPO); diphenyl (2,4,6-trimethylbenzoyl)-phosphine oxide (TPO); ethyl-2,4,6-trimethylbenzoylphenylphosphinate (ETP) (Lucirin TPO-L); diethyl benzoylphosphonate (DEBP); 2,2-dimethoxy-2-phenylacetonphenone (DMPA); benzoin methyl ether (BME); 2-hydroxy-2-methyl-1-phenyl-1-propanone (HMPP); bis(η 5-2,4-cyclopentadien-1-yl) bis[2,6-difluoro-3-(1H-pyrrol-1-yl) phenyl]titanium (Ti).

Remarkably, the high reactivity of these initiating systems based on (TMS)_3_Si^•^ allows the polymerization in rather hard conditions: (i) polymerization of monomers with low reactivity (e.g., epoxidized soybean oil (ESO) as a renewable epoxy monomer) and (ii) under very soft irradiations conditions (sunlight, daylight, fluorescence bulbs, LED bulbs; light intensity <10 mW/cm²) [70].

**Figure 3:**
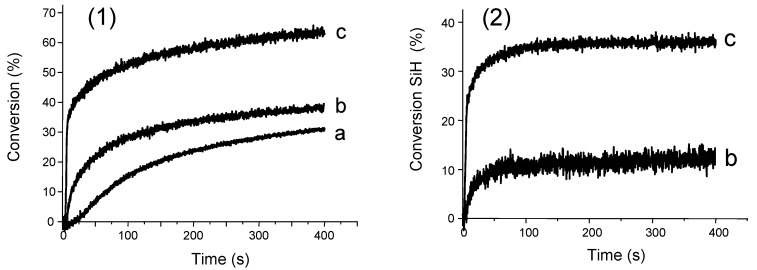
(**1**) Polymerization profiles of (3,4-epoxycyclohexane)methyl 3,4-epoxycyclohexyl-carboxylate (Uvacure 1500 from Cytec) under air. Upon a diode laser irradiation (405 nm) in the presence of (a) phenyl bis(2,4,6-trimethyl benzoyl)/Ph_2_I^+^ (1%/1% w/w), (b) phenyl bis(2,4,6-trimethyl benzoyl)/tetraphenyldisilane/Ph_2_I^+^ (1%/3%/1% w/w), and (c) phenyl bis(2,4,6-trimethyl benzoyl)/(TMS)_3_Si-H/Ph_2_I^+^ (1%/3%/1% w/w). (**2**) Conversion of the SiH content in the cases of (b) and (c).

From the different systems investigated, (TMS)_3_SiH led to the best polymerization profiles (e.g., see Figure 3-1, curve b for tetraphenyldisilane *vs.* curve c for (TMS)_3_SiH). For tetraphenyldisilane, a much lower Si-H conversion is observed compared to (TMS)_3_SiH (Figure 3-2). This can be ascribed to the higher bond dissociation energy BDE(Si–H) of this compound rendering the processes (15) and (13) less efficient. The low BDE(Si–H) for (TMS)_3_SiH associated with the fast oxidation process for the corresponding silyl radical ensures an efficient formation of silylium cations as ring opening initiating structures.

The high reactivity of silyliums with epoxy is associated with the formation of a strong Si–O bond. The interaction energy between (TMS)_3_Si^+ ^and cyclohexeneoxide is found 70 kJ/mol higher than the interaction of cations derived from carbon centered radicals (dimethoxybenzyl, ketyl or aminoalkyl) and cyclohexeneoxide [78]. This high affinity of silyliums with oxygen ensures a high reactivity for these structures in full agreement with the excellent polymerization profiles obtained above. 

### 4.5. (TMS)_3_SiH in Photoredox Catalysis: Polymerization Under Very Soft Irradiation Conditions

**Scheme 24:**
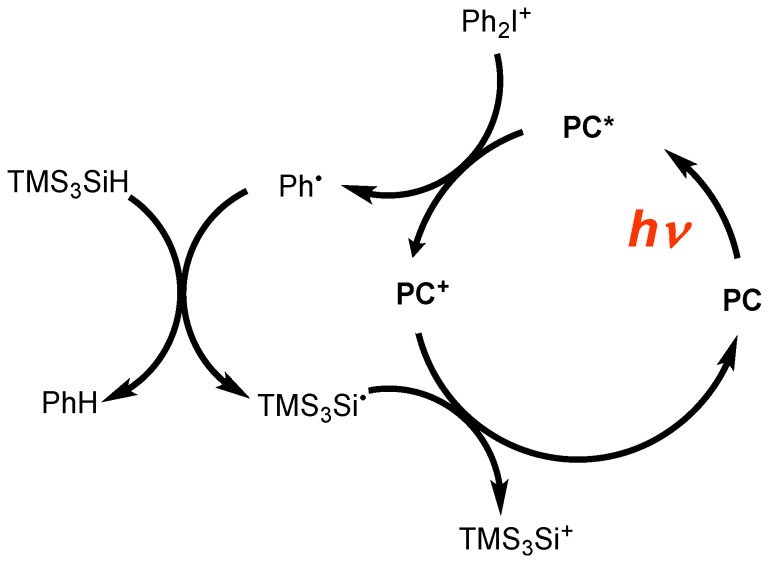
Photoredox catalysis using (TMS)_3_SiH.

The use of soft irradiations conditions (sunlight, household green fluorescent bulb, or LED bulbs) in ring opening photopolymerizations under air requested the development of very sensitive systems: this is associated with the very low light intensity available for such processes. Based on a recent approach called *photoredox catalysis* in organic chemistry for the formation of carbon centered radicals with such irradiation devices [92,93,94], this concept has been extended for the formation of (TMS)_3_Si^•^ and (TMS)_3_Si^+^ [95,96]. The catalytic cycle is depicted in Scheme 24.

**Figure 4:**
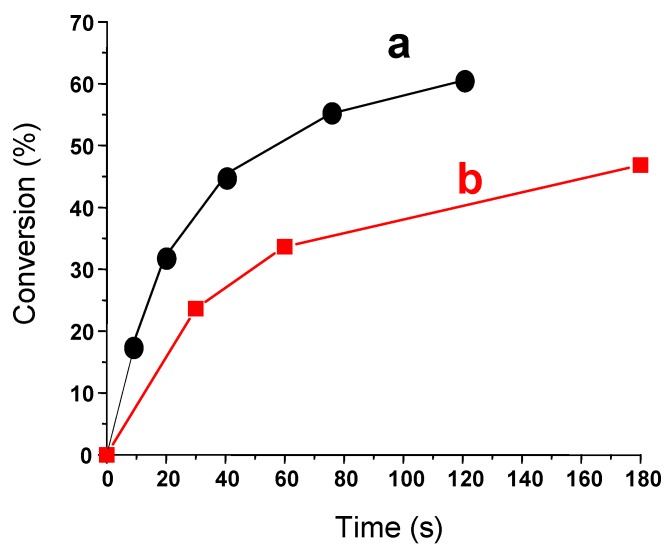
Polymerization profiles of (3,4-epoxycyclohexane)methyl 3,4-epoxycyclohexyl-carboxylate (Uvacure 1500 from Cytec) under air in the presence of (a) Ir(ppy)_3_/(TMS)_3_Si-H/Ph_2_I^+^ (0.2%/3%/2% w/w) upon a blue LED bulb; (b) Ru(phen)_3_^2+^/(TMS)_3_Si-H/Ph_2_I^+^ (0.2%/3%/2% w/w) upon a white LED bulb irradiation.

These new photoinitiated systems are based on a combination of photocatalysts (PC) (usually ruthenium or iridium complexes like tris(2,2′-bipyridine)ruthenium(II) dichloride hexahydrate Ru(bpy)_3_^2+^ and tris(2-phenylpyridine)iridium Ir(ppy)_3_), diphenyl iodonium salt and (TMS)_3_SiH. Good to excellent polymerization profiles are obtained (Figure 4).

### 4.6. (TMS)_3_SiH As a New Additive for Thermal FRP Processes

**Figure 5:**
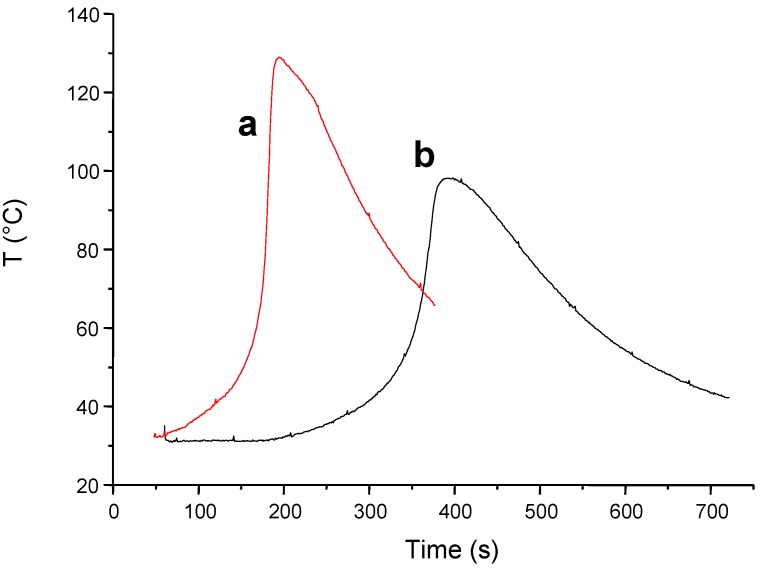
Polymerization process of an acrylate matrix (Ebecryl 605 from Cytec) initiated at RT under air for different initiating systems: (**a**) Cumene hydroperoxide/Cobalt (II) salt/(TMS)_3_SiH; (**b**) Cumene hydroperoxide/Cobalt (II) salt.

(TMS)_3_SiH can also be an excellent additive for thermal polymerization processes. The progress of the exothermic polymerization of acrylate can be followed by monitoring the sample temperature using a thermocouple and a published procedure [97]. This is well evidenced in Figure 5 where the addition of (TMS)_3_SiH to a classical Co(II)/hydroperoxide [98] initiating system results in a much faster polymerization in aerated conditions (curve a *vs.* curve b). This behavior is in full agreement with the ability of (TMS)_3_SiH to convert peroxyls to new initiating (TMS)_3_Si^•^ (reaction 6).

### 4.7. (TMS)_3_SiH Initiating Systems for Thermal Ring Opening Polymerization Processes

Recently, new initiating systems based on (TMS)_3_SiH for the thermal cationic polymerization of epoxides and vinyl ether derivatives were proposed [97]. They are based on a (TMS)_3_SiH/silver salt interaction which ensures a good to excellent polymerization at RT. Interestingly, silver nanoparticles Ag(0) (AgNP) are also formed *in situ*. For these systems, the formation of a silylium antimonate through the interaction of the silane with silver antimonate is found (reactions (16) and (17)) [99,100,101]. Free radicals were not involved and an ionic mechanism was assumed. The good polymerization ability of the (TMS)_3_SiH/AgSbF_6_ system appears here to be in full agreement with the formation of (TMS)_3_Si^+^SbF_6_^−^ and H^+^SbF_6_^−^, which are largely known [59,60,61] as efficient initiating species for both thermal and photochemical cationic polymerization of EPOX and DVE-3: 


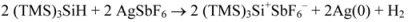
(16)


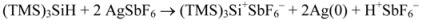
(17)

## 5. (TMS)_3_SiH As a Model for H-Si Surface

Silicon is clearly the most important material used for microelectronic applications. The preparation of ideal hydrogen-terminated silicon surface (H-Si) has attracted much interest in recent years, as they constitute an excellent potential substrate for nanotechnology [3,4,5]. The two flat surfaces H-Si (111) and H-Si (100)-2 × 1 resemble (Me_3_Si)_3_SiH in that the three silicon atoms are attached at the SiH moieties. The Si-H bonds can serve as chemical handles, which allow to introduce new functionalizations. Several (Me_3_Si)_3_SiH reactions, in particular activated alkenes and alkynes, have been adopted and applied to surfaces, and that mechanistic schemes are often proposed in analogy with (Me_3_Si)_3_SiH radical chemistry [102,103]. It was shown that radical cations generated by photoinduced electron transfer can react with different nucleophiles used for monolayer formation onto H-Si. The reaction rate constants for a series of nucleophiles, which were selected based on their relevance for monolayer formation onto H-terminated silicon surfaces have been described [104,105,106]. Interestingly, from these reaction rate constants it is clearly found that 1-alkynes react approximately twice faster with silyl radical cations than to 1-alkenes, a relationship related to the faster monolayer formation of 1-alkynes [107,108]. It was also shown that acids, water, aldehydes and alcohols react also significantly faster with silyl radical cations.

## 6. Conclusions

The application of (TMS)_3_SiH radical-based reactions in the last decade has expanded considerably. (TMS)_3_SiH has become popular among synthetic, polymer and material chemists indicating its multidisciplinary role. Unique transformations are possible, allowing one to generate structures that would otherwise be very difficult to synthesize. (TMS)_3_SiH is an additive of newly developed photoinitiating systems in radical and radical-promoted cationic polymerization. (TMS)_3_SiH and its reactivity represent a model for the functionalization of silicon surfaces using radical chemistry. We trust that this review will serve as a platform to expand (TMS)_3_SiH radical chemistry with new and exciting discoveries.
